# Clinical and neuroimaging review of triplet repeat diseases

**DOI:** 10.1007/s11604-022-01343-5

**Published:** 2022-09-28

**Authors:** Ryo Kurokawa, Mariko Kurokawa, Akihiko Mitsutake, Moto Nakaya, Akira Baba, Yasuhiro Nakata, Toshio Moritani, Osamu Abe

**Affiliations:** 1 Department of Radiology, Graduate School of Medicine, The University of Tokyo, 7-3-1, Hongo, Bunkyo-ku, Tokyo, 113-8655 Japan; 2 Division of Neuroradiology, Department of Radiology, University of Michigan, 1500 E Medical Center Dr, Ann Arbor, MI 48109 USA; 3 Department of Neurology, International University of Health and Welfare, Mita Hospital, 1-4-3 Mita, Minato-ku, Tokyo, 108-8329 Japan; 4 Department of Neuroradiology, Tokyo Metropolitan Neurological Hospital, 2-6-1 Musashidai, Fuchu, Tokyo 183-0042 Japan

**Keywords:** Triplet repeat disease, Neurodegeneration, Computed tomography, Magnetic resonance imaging

## Abstract

Triplet repeat diseases (TRDs) refer to a group of diseases caused by three nucleotide repeats elongated beyond a pathologic threshold. TRDs are divided into the following four groups depending on the pathomechanisms, although the pathomechanisms of several diseases remain unelucidated: polyglutamine disorders, caused by a pathologic repeat expansion of CAG (coding the amino acid glutamine) located within the exon; loss-of-function repeat disorders, characterized by the common feature of a loss of function of the gene within which they occur; RNA gain-of-function disorders, involving the production of a toxic RNA species; and polyalanine disorders, caused by a pathologic repeat expansion of GCN (coding the amino acid alanine) located within the exon. Many of these TRDs manifest through neurologic symptoms; moreover, neuroimaging, especially brain magnetic resonance imaging, plays a pivotal role in the detection of abnormalities, differentiation, and management of TRDs. In this article, we reviewed the clinical and neuroimaging features of TRDs. An early diagnosis of TRDs through clinical and imaging approaches is important and may contribute to appropriate medical intervention for patients and their families.

## Introduction

More than half of the human genome consists of repetitive DNA sequences, a large number of which lie within genes and their regulatory regions [1]. There are over 1 million discrete loci for short tandem repeats (1–6 base pair motifs) in the human genome, accounting for approximately 3% of genomic DNA [2]. These repetitive DNAs of various lengths provide evolutionary plasticity to adapt to different environments in humans. However, when the repetition exceeds a pathologic threshold, the gene expression and/or function of the gene product is altered, resulting in undesired conditions.

Triplet repeat diseases (TRDs) refer to a group of diseases caused by three nucleotide repeats elongated beyond a pathologic threshold [3]. In 1991, two different research groups successively discovered a CAG expansion in the exon of the androgen receptor gene in X-linked spinal and bulbar muscular atrophy (SBMA) [4] and a CGG expansion in the 5’ untranslated region of the fragile X mental retardation 1 (*FMR1*) gene in fragile X syndrome (FXS) [5]. In 2019, three diseases (neuronal intranuclear inclusion disease [NIID], oculopharyngeal myopathy with leukoencephalopathy [OPML], and oculopharyngodistal myopathy-1 [OPDM1]) were newly recognized as TRDs [6], and now there are more than 20 TRDs with frequent abnormal neuroimaging findings. TRDs are divided into the following four groups, depending on the pathomechanisms, although the pathomechanisms of several diseases remain unelucidated [7]:

- Polyglutamine (polyQ) disorders: all diseases of this group share the common feature of being caused by pathologic repeat expansion of CAG (coding the amino acid glutamine) located within the exon.
- Loss-of-function repeat disorders: this group includes disorders with different combinations of nucleotide repeats in various gene locations, but shares the common feature of a loss of function of the gene within which they occur.
- RNA gain-of-function disorders: all diseases of this group share the common feature of involvement in the production of a toxic RNA species.
- Polyalanine (polyA) disorders: all diseases of this group share the common feature of being caused by pathologic repeat expansion of GCN (coding the amino acid alanine) located within the exon.

Many of these TRDs manifest through neurologic symptoms, and neuroimaging, especially brain magnetic resonance imaging (MRI), plays a pivotal role in the detection of abnormalities, differentiation, and management of TRDs. TRD diagnosis is often challenging without genetic testing, due to the unspecific imaging findings; moreover, various degrees of clinical and imaging overlaps exist between TRDs or non-TRDs. Nevertheless, some patients with TRDs present pathognomonic imaging features and/or clinical pictures. In this article, we reviewed the clinical and neuroimaging characteristics of TRDs.

### PolyQ disorders

PolyQ disorders include the following diseases: Huntington disease, dentatorubural-pallidoluysian atrophy (DRPLA), spinocerebellar ataxia type 1 (SCA1), SCA2, SCA3/Machado-Joseph disease (MJD), SCA6, SCA7, SCA17, and SBMA. Although the pathogenetic mechanism of PolyQ disorders has not been fully understood, most evidence suggests that abnormally elongated CAG repeats generate mutant proteins with abnormally elongated polyglutamates, which cause neurotoxicity through an abnormal conformation and misfold [7, 8]. With the exception of X-linked SBMA, the inheritance pattern of PolyQ disorders is autosomal dominant, and many polyQ disorders display “anticipation”, which is defined as more enhanced disease severity in successive generations of patients with more elongated CAG repeats and earlier disease onset [9].

### Huntington disease

**Fig. 1:**
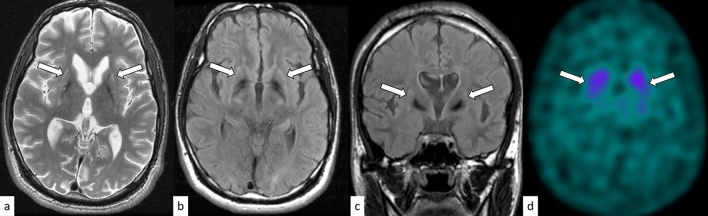
A 24-year-old man with Huntington disease. T2-weighted axial image **a**, FLAIR axial image **b**, and FLAIR coronal image **c** show hyperintensity and atrophy of the bilateral caudate nuclei and putamima, and atrophy of the globi pallidi (arrows). Decreased DAT uptake with a specific binding ratio of 2.12 (right striatum) and 2.09 (left striatum) is observed on DaTscan (**d**)

Huntington disease is an autosomal dominant disorder involving motor, cognitive, and psychiatric disturbances. Huntington disease is caused by an abnormal expansion of CAG repeats (full-penetrance pathogenic repeat number [same hereafter]: ≥ 39) in the Huntingtin (*HTT*) gene on 4p16.3 [10]. The mean age of onset is between 35 and 44 years, although the juvenile form with an early onset of symptoms (< 20 years) accounts for 5–10% of cases. The prevalence of Huntington disease is 5.7 per 100,000 and 0.4 per 100,000 in the Caucasian and Asian populations, respectively [11]. The characteristic motor symptoms include choreatic movements, hypokinesia, akinesia, and rigidity [12]. Cognitive decline can be seen long before the motor symptoms, while it can also be mild in later stages. Moreover, psychiatric symptoms, such as depression, anxiety, and irritability, are frequently present. Anticipation occurs more commonly in the paternal transmission of the mutated allele, with mean intergenerational expansions of CAG repeats of + 7.3 and + 0.7 with paternal and maternal transmissions, respectively [13]. On MRI, Huntington disease is characterized by progressive striatal (pallidum, putamen, and caudate) atrophy with T2 hyperintensity, which is the largest and earliest change in the subcortex (Fig. 1) [14], and correlates with the age of onset, disease duration, and CAG repeat length [15]. Other MRI findings include cortical thinning, which progresses from posterior to anterior cortical regions [15]. Altered dopamine signaling is demonstrated by reduced striatal dopamine transporter (DAT) binding on positron emission tomography (PET)/single photon emission computed tomography (SPECT) [15]. Inherited diseases with bilateral caudate atrophy, such as neuroacanthocytosis, McLeod syndrome, and SCA17 are imaging differential diagnoses of Huntington disease [10].

### DRPLA

**Fig. 2:**
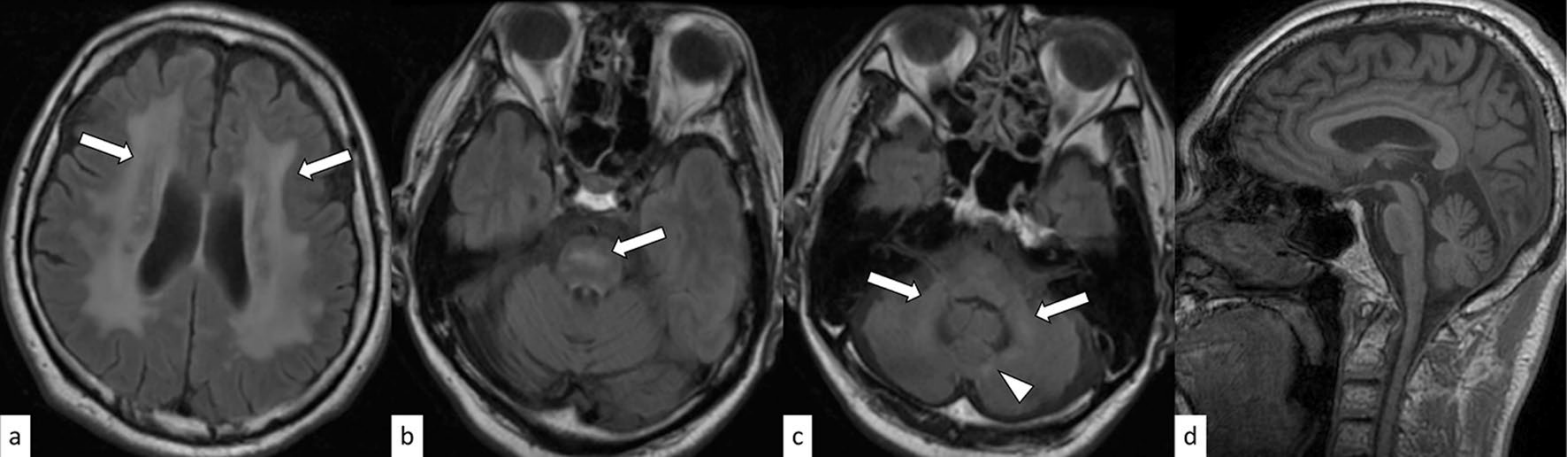
A 53-year-old man with DRPLA. FLAIR axial images show hyperintensity in the supratentorial white matter (**a**, arrows), pons (**b**, arrow), and cerebellar white matter (**c**, arrows). FLAIR hyperintensity in the paravermal area is observed (**c**, arrowhead). T1-weighted sagittal image shows atrophic brainstem (**d**)

DRPLA is an autosomal dominant disorder caused by an abnormal expansion of CAG repeats (48–93) in the Atrophin 1 (*ATN1*) gene on 12p13.31 [16]. The mean age of onset is 31.5 years, and the clinical manifestations vary depending on the age of onset. The prevalence of DRPLA is 0.2–0.7 per 100,000 in the Japanese population, whereas it is rare in other countries [17]. The characteristic manifestations include ataxia, progressive myoclonus epilepsy, seizures, and progressive intellectual deterioration in patients with disease onset before 20 years of age, while ataxia, choreoathetosis, dementia, and psychiatric disturbance are present in those with disease onset after 20 years of age [16]. On MRI, only cerebellar atrophy can be seen in the early stages; however, as the disease progresses, atrophy of the brainstem (particularly the pontine tegmentum) and cerebrum accompanies it. The age at MRI and CAG repeat length correlates with the atrophic changes [16]. Hyperintensity on fluid-attenuated inversion recovery (FLAIR) images is observed in the cerebral white matter, cerebellar white matter, thalamus, and brainstem, in a decreasing order (Fig. 2) [18]. FLAIR hyperintensity in the cerebellar paravermal area, which was initially reported as the characteristic sign in NIID and fragile X-associated tremor/ataxia syndrome (FXTAS) [19, 20], can also be present in DRPLA [18].

### SCA1

**Fig. 3:**
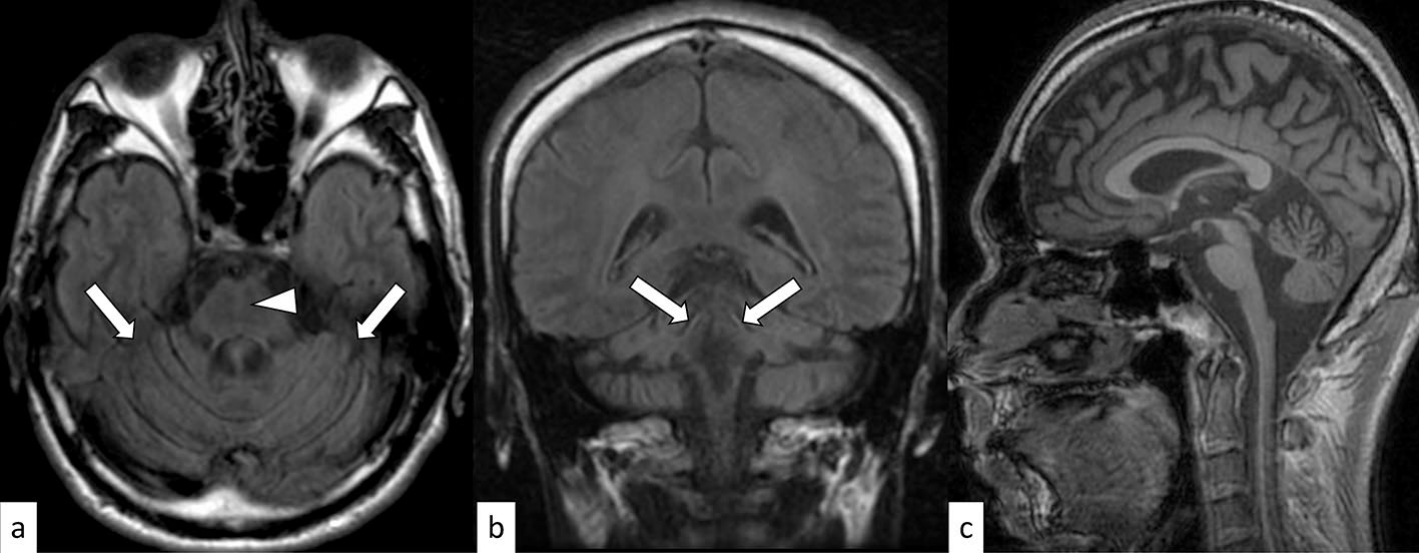
A 48-year-old man with SCA1. FLAIR axial image **a** and FLAIR coronal image **b** show cerebellar atrophy including superior cerebellar peduncles (arrows) and a vertical hyperintense line reflecting pontine atrophy (an incomplete hot cross bun sign) (**a**, arrowhead). T1-weighted sagittal image shows an atrophy of the brainstem and cerebellum (**c**)

SCA1 is an autosomal dominant disorder caused by an abnormal expansion of CAG repeats (≥ 39) in the Ataxin 1 (*ATXN1*) gene on 6p22.3 [21]. The onset is typically in the third or fourth decade of life, and gait ataxia is the initial symptom in 88% of cases; moreover, all patients eventually display cerebellar dysfunction [21, 22]. In addition to gait ataxia and dysarthria, SCA1, SCA2, and SCA3/MJD share clinical manifestations such as pyramidal involvement, peripheral neuropathy, and intellectual impairment [23]. Given the variety of common symptoms and varying degrees of severity, it is often challenging to distinguish SCA1 from other hereditary spinocerebellar degenerations without known significant family history. Computed tomography (CT) and MRI show atrophy in the cerebellum and brainstem, as well as gray matter atrophy in the supratentorial cortex, putamen, and caudate (Fig. 3) [24]. The hot cross bun sign, composed by hyperintensity of the cerebellar white matter, middle cerebellar peduncles, and pons on T2-weighted imaging (T2WI), while sparing the pyramidal tracts and medial lemnisci, has been infrequently reported in SCA1 [25–27]. Cerebellar atrophy predominantly involves the sensorimotor lobules (V, VI, and VIII) and the high-order cognitive processing lobules (VI, crus II, VIIB, and VIIIA) during the early symptomatic phase [24].

### SCA2

**Fig. 4:**
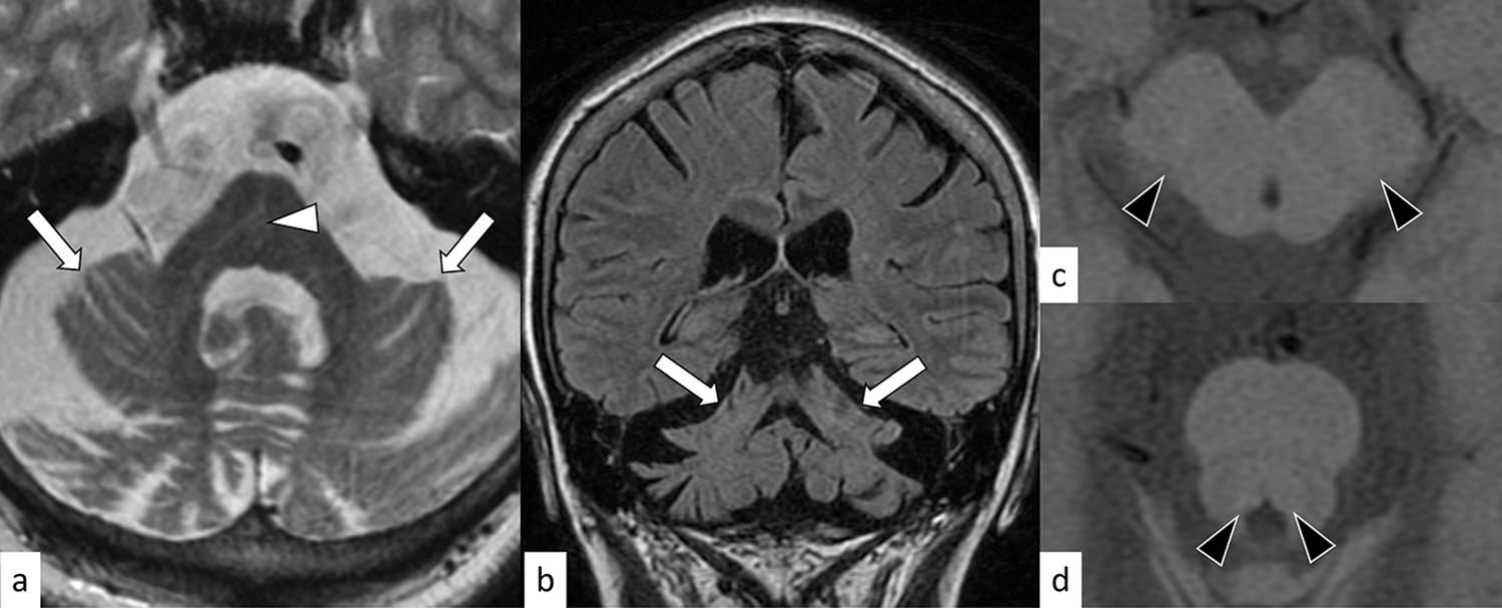
A 33-year-old woman with SCA2. T2-weighted axial image **a** and FLAIR coronal image **b** show severe cerebellar atrophy (arrows). The hot cross bun sign is also observed (**a**, white arrowhead). Decreased melanin deposition in the bilateral substantia nigra **c** and locus ceruleus **d** is present on neuromelanin images (black arrowheads)

SCA2 is an autosomal dominant disorder caused by an abnormal expansion of CAG repeats (≥ 33) in the Ataxin 2 (*ATXN2*) gene on 12q24.12 [23]. The age at onset is usually in the fourth decade, and gait ataxia is the initial symptom in 87% of cases [22]. In addition to cerebellar dysfunction, extrapyramidal signs such as bradykinesia, postural/resting tremor, and focal/segmental dystonia as well as reduced saccadic velocity are frequently observed in SCA2 [23, 28]. MRI shows cerebellar and brainstem atrophy. Hot cross bun sign can be observed in 25.7–29.4% of the patients with SCA2 (Fig. 4) [25, 29, 30]. Decreased striatal uptake of DAT tracers on PET/SPECT has been reported in symptomatic SCA2 with parkinsonism, consistent with nigrostriatal dysfunction secondary to substantia nigra degeneration [30]. Furthermore, signal loss on neuromelanin-sensitive MRI in the substantia nigra and locus ceruleus has been reported in SCA2, although its diagnostic performance for differentiation from other hereditary spinocerebellar degenerations remains unclear [31].

### SCA3/MJD

**Fig. 5:**
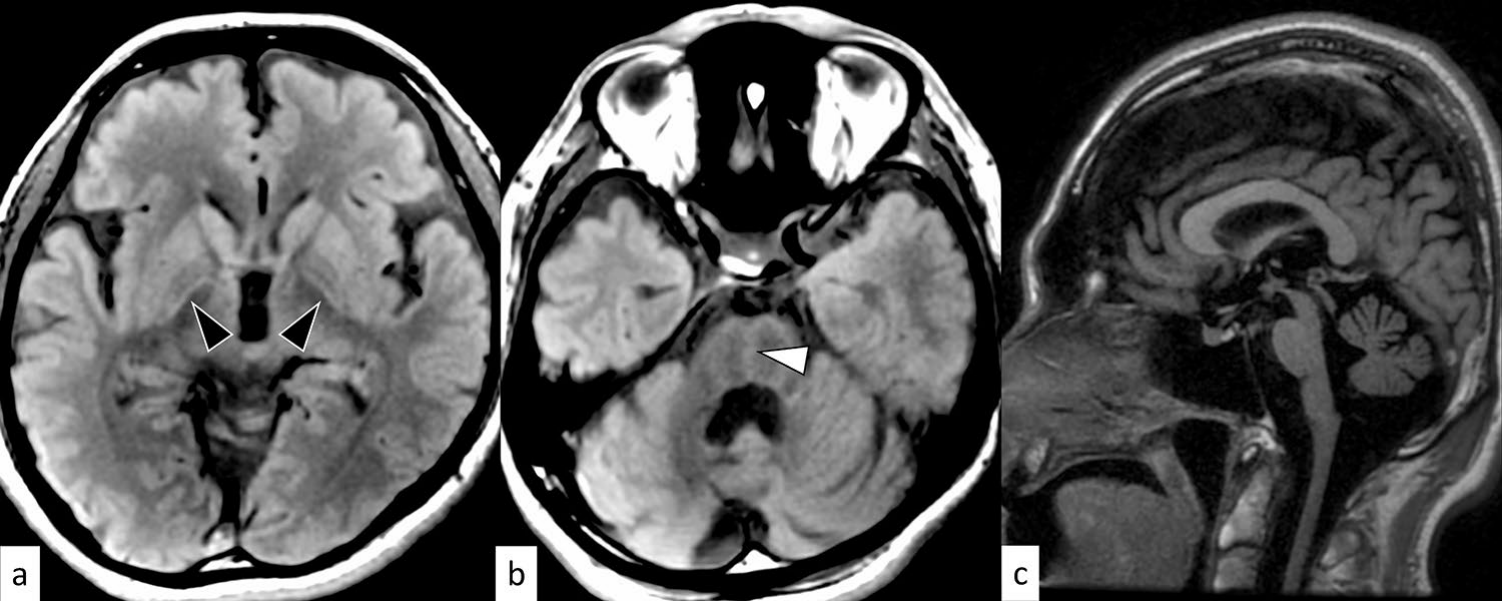
A 41-year-old man with SCA3/MJD. Proton-weighted axial images show hyperintensity bands in the bilateral medial segments of the globi pallidi (**a**, black arrowheads) and an incomplete hot cross bun sign (**b**, white arrowhead). T1-weighted sagittal image shows an atrophy of the brainstem and cerebellum (**c**)

SCA3/MJD is an autosomal dominant disorder caused by an abnormal expansion of CAG repeats (≥ 45) in the Ataxin 3 (*ATXN3*) gene on 14q32.12 [32]. The mean age at onset is the middle 30 s, and gait ataxia is the initial symptom in 85% of cases [22]. Highly specific clinical features of SCA3/MJD include bulging eyes and action-induced facial and lingual fasciculations. The bulging eyes reflect upper eyelid retraction and are not due to eyeball protrusion as seen in thyroid ophthalmoplegia [33]. Atrophy has been reported on MRI over a wide range of regions, including the cerebral cortex, thalamus, basal ganglia, cerebellum, and brainstem (Fig. 5). However, atrophy is initially observed in the cerebellum and brainstem and eventually extends supratentorially [34]. Atrophy of the basal ganglia is observed in patients with long disease duration (> 10 years) [34]. The hot cross bun sign is present in 1.3% of patients [29]. Hyperintensity on proton density-weighted images of the internal segment of the globus pallidus can be a clue to differentiate SCA3/MJD from DRPLA, which shows hyperintensity in the external segment of the globus pallidus.

### SCA6

**Fig. 6:**
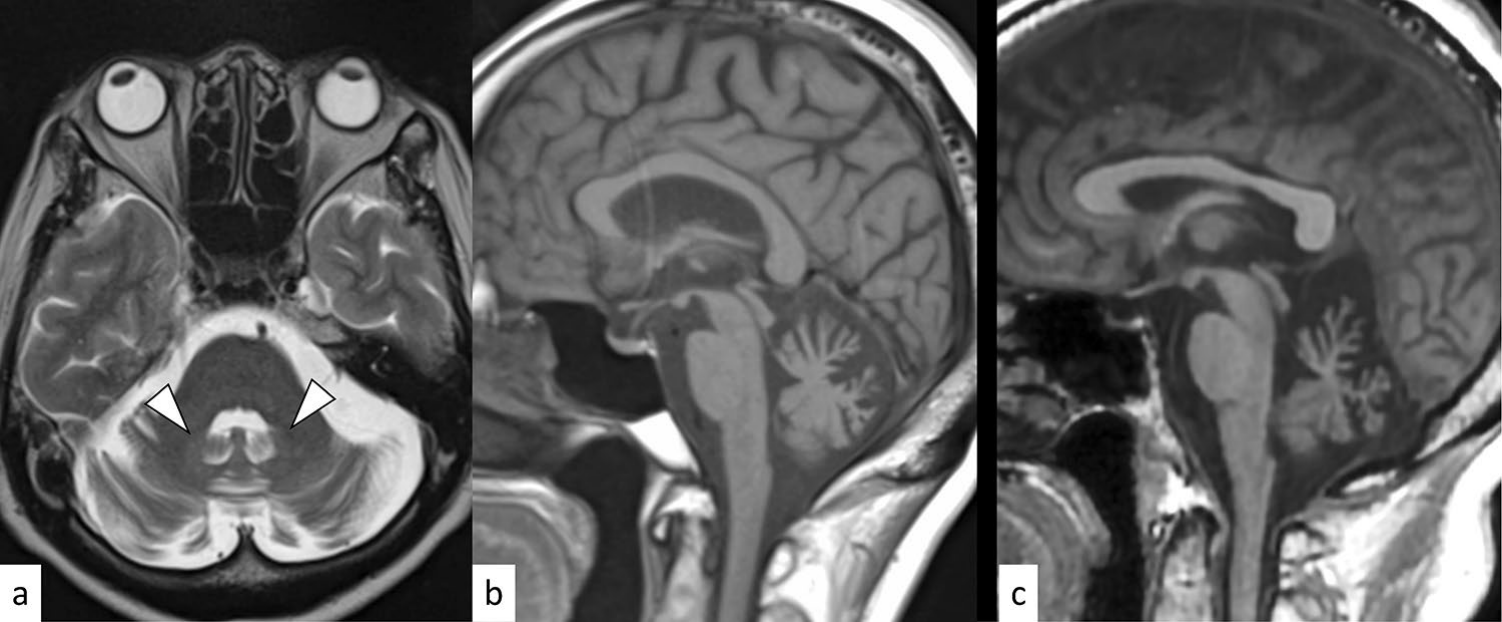
A 64-year-old woman with SCA6 (**a**, **b**) and a 50-year-old woman with familial hemiplegic migraine type 1 (**c**). T2-weighted axial image shows the absence of T2 hypointensity in the bilateral dentate nuclei (**a**, white arrowheads). The hot cross bun sign is absent (**a**). Pure cerebellar atrophy is observed on T1-weighted sagittal images in both patients (**b**, **c**)

SCA6 is an autosomal dominant disorder caused by an abnormal expansion of CAG repeats (20–33) in the *CACNA1A* gene on 19p13.13 [35]. Anticipation has not been observed in SCA6 [35]. The mean age of onset is between 43 and 52 years, and gait ataxia is the initial symptom in 76% of cases [22]. The incidence of downbeat positioning nystagmus (84%) is much higher in SCA6 than in other forms of SCA [36]. Other diseases with *CACNA1A* gene mutation include episodic ataxia type 2 and familial hemiplegic migraine 1 (Fig. 6); some patients with SCA6 show clinical overlap with these diseases. MRI shows progressive cerebellar atrophy including the dentate nuclei (Fig. 6) [37]. Normal T2 hypointensity in the dentate nuclei may be absent in SCA6 [37]. The absent T2 hypointensity in the dentate nuclei in SCA6 may be attributable to the decreased iron deposition, as indicated by quantitative susceptibility mapping studies [38, 39].

### SCA7

**Fig. 7:**
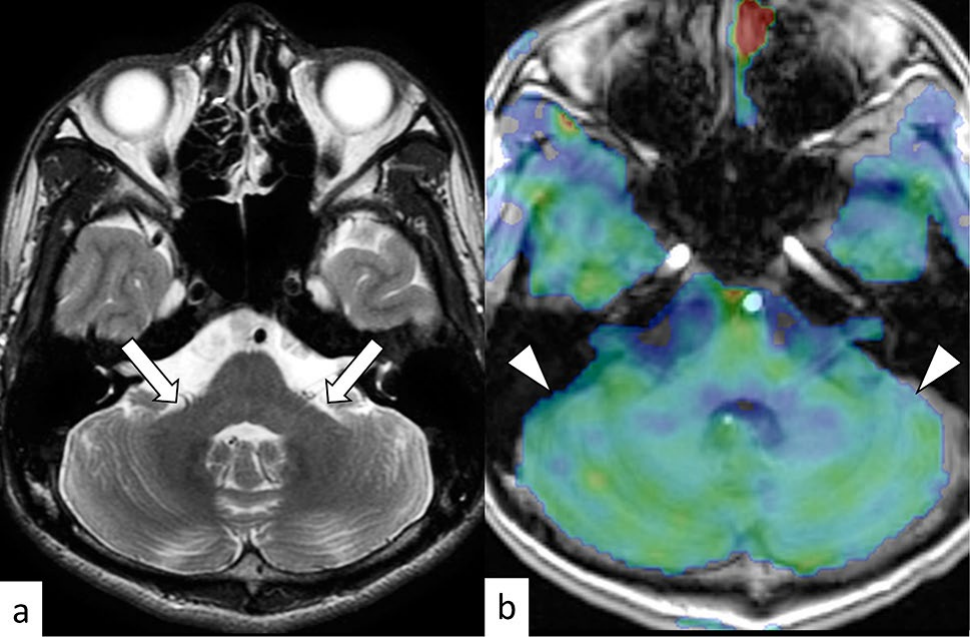
A 21-year-old woman with SCA7. T2-weighted axial image shows mild cerebellar atrophy (**a**, arrows). Arterial spin labeling image shows decreased blood flow in the cerebellum (**b**, arrowheads)

SCA7 is an autosomal dominant disorder caused by an abnormal expansion of CAG repeats (≥ 34) in the Ataxin 7 (*ATXN7*) gene on 3p14.1 [40]. SCA7 is characterized by cerebellar ataxia with progressive vision impairment due to retinal degeneration. This combination of symptoms is pathognomonic for SCA7; however, because of the extremely excessive anticipation, affected pediatric patients may develop the symptoms before their parents or grandparents [40]. Furthermore, the genetic diagnosis of pediatric patients can be a pre-onset diagnosis for one of their parents. In addition to the retina, an abnormal aggregation of *ATXN7* is found in the cerebral cortex, basal ganglia, thalamus, midbrain, pons, medulla oblongata, and cerebellum [41]. In infantile cases, which progress more rapidly and have a poorer prognosis, abnormal protein aggregation leading to cellular degeneration is not limited to neural tissues but also involves non-neural tissues, including the anterior pituitary lobe, stomach, pancreas, adrenal glands, kidneys, intestines, cardiovascular tissues, and skeletal muscles [41]. On MRI, atrophy of the cerebellum and pons, and T2 hyperintensity of the middle cerebellar peduncles is occasionally observed (Fig. 7) [42, 43]. The hot cross bun sign is rare in patients with SCA7 [29].

### SCA17

**Fig. 8:**
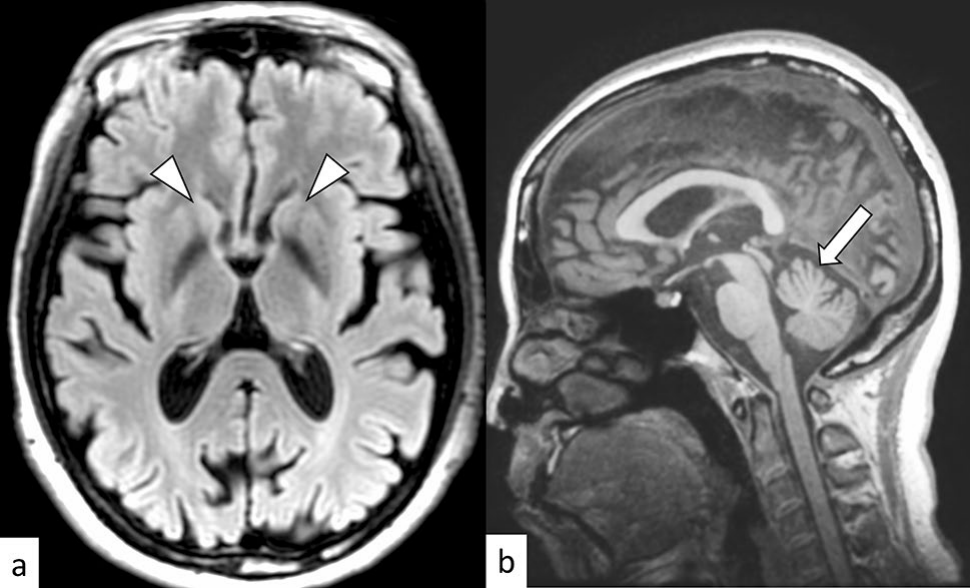
A 40-year-old woman with SCA17. FLAIR axial image shows atrophy of the bilateral caudate nuclei (**a**, arrowheads). T1-weighted sagittal image **b** shows cerebellar atrophy (arrows), while the brainstem is spared

SCA17, also known as Huntington disease-like 4 (HDL4), is an autosomal dominant disorder caused by an abnormal expansion of CAG repeats (41–66) in the TATA-binding protein (*TBP*) gene on 6q27 [44]. The mean age at onset is 34.6 years. Cerebellar ataxia and psychiatric symptoms are often the initial clinical manifestations of SCA17, followed by involuntary movements such as chorea and dystonia, parkinsonism, dementia, and pyramidal tract signs [44]. On MRI, atrophy of the cerebellum and caudate nucleus may be present, not only after disease onset, but also in pre-mutation carriers (Fig. 8) [45]. Significantly reduced glucose metabolism on ^18^F-FDG-PET in the caudate nucleus has been reported in symptomatic patients with SCA17 [45]. Differential diagnoses include autosomal dominant hereditary disorders with choreatic movements (i.e., Huntington disease, HDL1, HDL2, SCA1, SCA2, SCA3/MJD, DRPLA, neuroferritinopathy, and benign hereditary chorea) [23, 44].

### SBMA

**Fig. 9:**
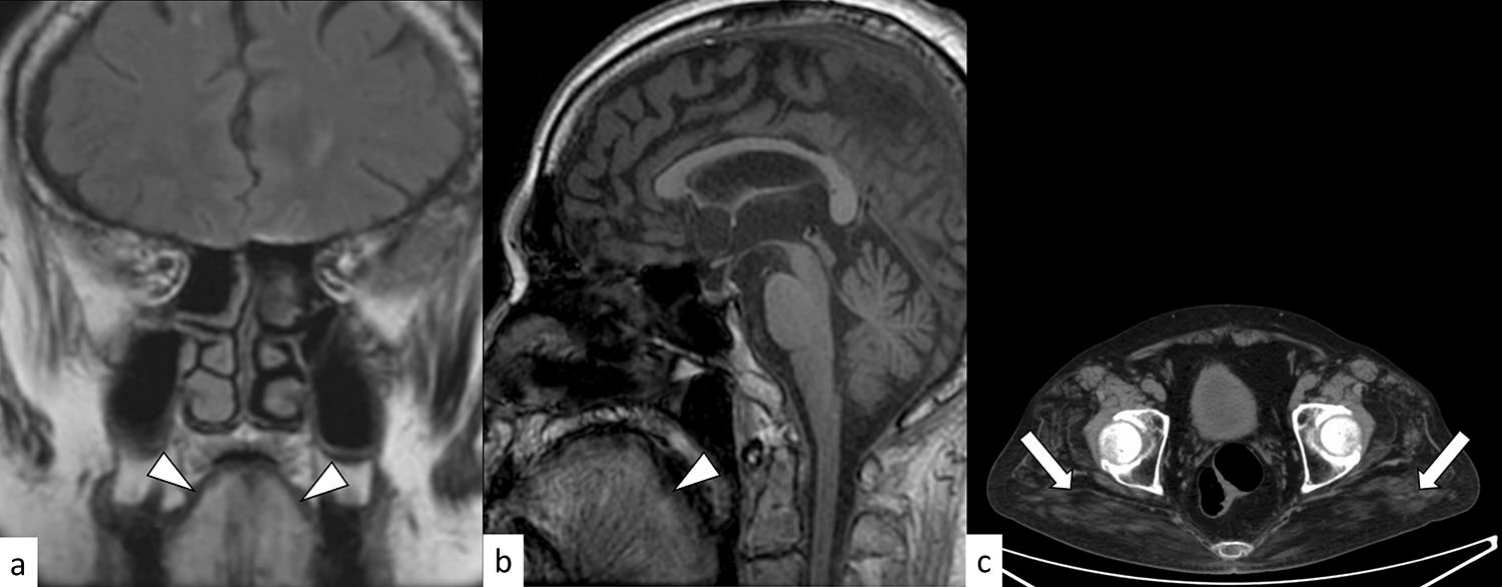
A 71-year-old man with SBMA. FLAIR coronal image **a** and T1-weighted sagittal image **b** show hyperintensity atrophic tongue muscles, suggestive of fat infiltration (arrowheads). Non-enhanced axial CT image shows fat infiltration in the bilateral gluteal muscles (**c**, arrows)

SBMA, also known as Kennedy’s disease, is an X-linked gradually progressive neuromuscular disorder caused by an abnormal expansion of CAG repeats (≥ 38) in the androgen receptor gene [46]. The mean age of onset is the mid-40 s, and the most common symptoms are weakness, tremor, and cramping [46]. SBMA is characterized by androgen insensitivity including sexual dysfunction, gynecomastia, and testicular atrophy, which may be apparent before motor involvement. Furthermore, bulbar manifestations, including dysarthria, nasal speech, and tongue atrophy, can present early in the disease course. Unlike amyotrophic lateral sclerosis, which most often mimics SBMA, upper motor neuron signs are usually absent [46]. Skeletal MRI and CT show fat infiltration in bulbar and limb muscles (Fig. 9) [47]. Miyata et al. reported that facial nerve atrophy and a small cochlear nerve to facial nerve ratio could be observed in the early stage of SBMA [48].

### Loss-of-function repeat disorders

Loss-of-function repeat disorders include the following diseases: FXS, fragile XE syndrome (FRAXE), and Friedreich’s ataxia (FRDA). Patients with these diseases have unstable triplet repeat expansions in non-protein-coding regions of genes; the expansion is in the 5’ untranslated region in FXS and FRAXE, whereas the expansion is in the first intron in FRDA [49]. The expanded repeat causes transcriptional silencing (loss-of-function) of the genes in these diseases [49].

### FXS

**Fig. 10:**
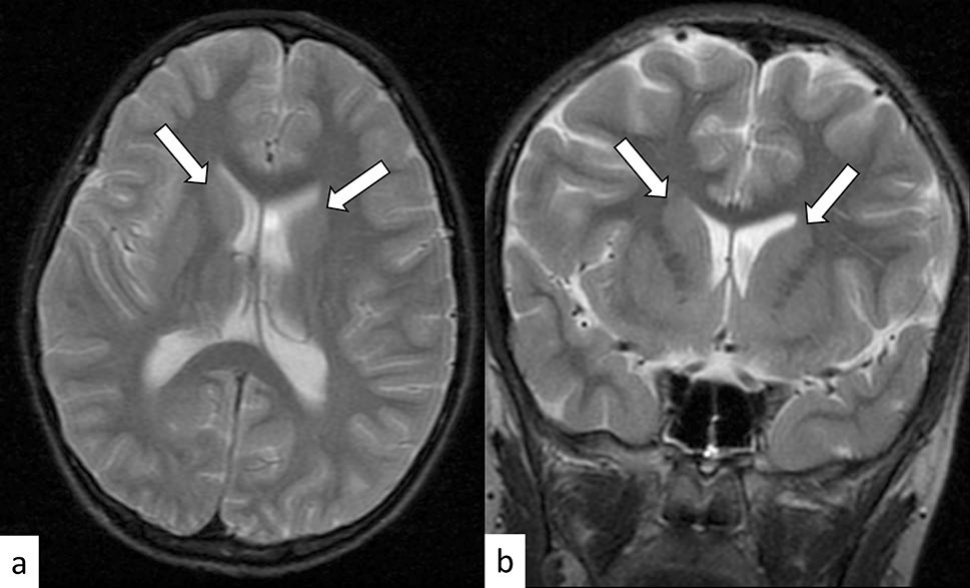
A 4-year-old girl with fragile X syndrome. T2-weighted axial **a** and coronal **b** images show slightly enlarged heads of the caudate nuclei (arrows)

FXS is an X-linked disorder caused by an abnormal expansion of CGG repeats (> 200) in the *FMR1* gene on Xq27.3 [50]. The worldwide estimated prevalence of FXS is 1:5,000–7,000 males and 1:4,000–6,000 females; further, FXS is the second cause of intellectual disability after Down syndrome [51]. In FXS, severe and less severe intellectual disabilities are usually evident in males and females, respectively, with characteristic facial features such as large ears and elongated face, large testicles (boys), learning disabilities, and behavioral abnormalities (autism spectrum disorder and attention deficit/hyperactivity disorder) occurring frequently [50]. Other than facial, central nervous system (CNS), neuropsychiatric, musculoskeletal (e.g., joint laxity and flat feet), cardiovascular (e.g., mitral valve prolapse and aortic dilatation), and eye (e.g., refractive errors and strabismus) manifestations, as well as gastrointestinal reflux may occur [51]. Brain MRI shows caudate enlargement, which is specific for FXS in contrast to other neurodevelopmental disorders and can be observed within the first 3 years of life (Fig. 10) [52]. Other *FMR1*-related disorders include FXTAS and fragile X-associated primary ovarian insufficiency [50].

### FRAXE

FRAXE is an X-linked disorder caused by an abnormal expansion of CCG repeats (> 250) in the fragile X mental retardation 2 gene on Xq28 [53]. The estimated prevalence of FRAXE is 1:50,000–100,000 males [54]. Affected patients show mild (intelligence quotient 50–70) to borderline (intelligence quotient 70–85) mental retardation associated with learning disability, communication deficits, attention problems, hyperactivity, and autistic behavior [53]. Physical features characteristic of FXS are absent in FRAXE [50]. Although characteristic neuroimaging features of FRAXE have not yet been established, no abnormal finding has been reported in several cases [55, 56].

### FRDA

**Fig. 11:**
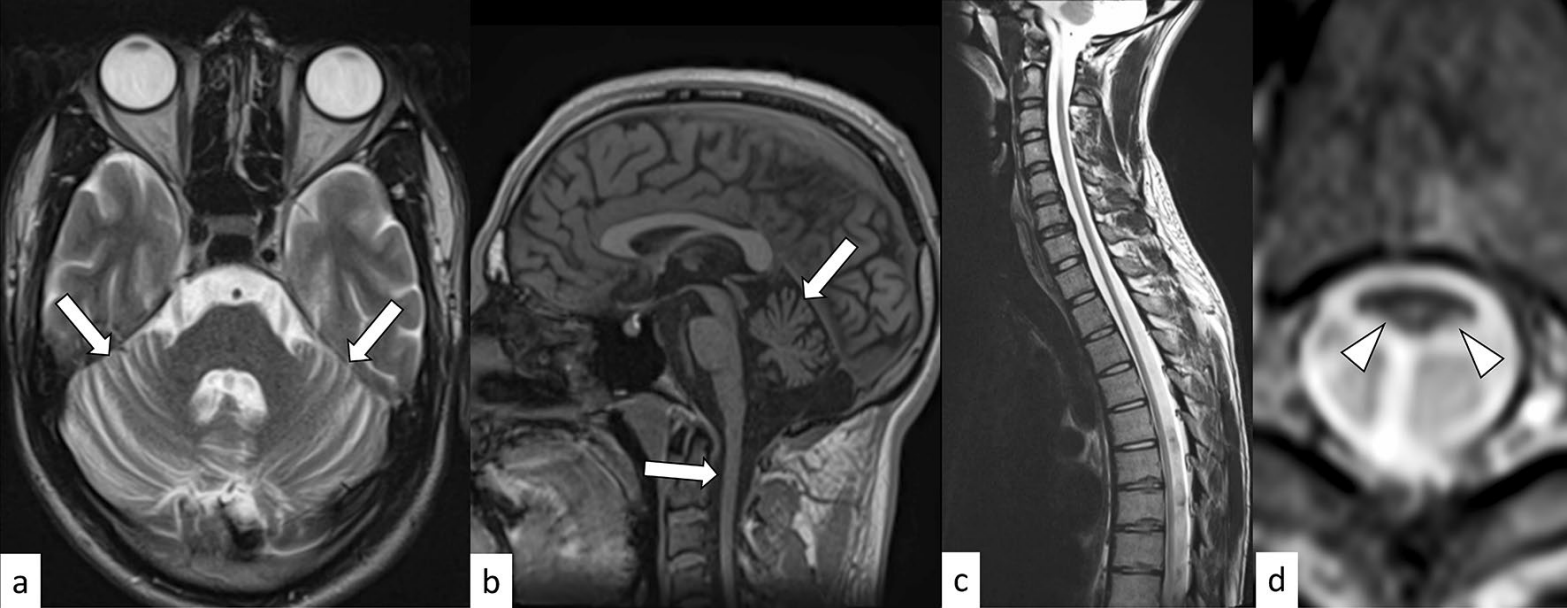
A 22-year-old man with Friedreich’s ataxia. T2-weighted axial image **a** and T1-weighted sagittal image **b** show atrophy of the cerebellum and brainstem (arrows). T2-weighted sagittal image shows diffuse spinal atrophy (**c**). T2-weighted axial image shows hyperintensity in the bilateral dorsolateral funiculi (**d**, arrowheads)

FRDA is an autosomal recessive disorder resulting from an abnormal expansion of GAA repeats (≥ 66) in the Frataxin (*FXN*) gene on 9q21.11 [57]. The prevalence of FRDA is 2–4:100,000 persons, and is the most common inherited ataxia in Europe, the Middle East, South Asia, and North Africa; nevertheless, FRDA has not been documented in Southeast Asians, sub-Saharan Africans, or Native Americans [57]. Typically, the disease onset occurs around puberty, and patients present with gait and limb ataxia, dysarthria, dysphagia, and scoliosis, and are wheelchair-bound by 10–15 years after onset. About two-thirds of the patients develop hypertrophic cardiomyopathy, and up to 30% have diabetes mellitus [57]. Neuropathological abnormalities occur predominantly in the dorsal root ganglia, dorsal horn of the spinal cord, cerebellar dentate nuclei, spinocerebellar tract, and corticospinal tract [58]. On MRI, atrophy is observed in the white matter of the midbrain, pons, medulla oblongata, and cerebellar peduncles, cerebellar lobules I–IV, and precentral gyrus (Fig. 11). Cases with early disease onset show an early degeneration of the cerebellar motor areas (lobules I–IV and VIIIb) and cerebellar peduncles [58]. The duration and severity of the disease correlate with atrophy of the dentate nucleus, brainstem, and superior and inferior cerebellar peduncles [58]. Differential diagnoses of FRDA include diseases involving pediatric cerebellar ataxia and dorsal funiculus degeneration, such as ataxia with vitamin E deficiency, abetalipoproteinemia, posterior column ataxia with retinitis pigmentosa, and ataxia-oculomotor apraxia type 1 [57, 59].

### RNA gain-of-function disorders

RNA gain-of-function disorders include the following diseases: myotonic dystrophy type 1 (DM1) and FXTAS. Expanded CTG repeats in the 3’ non-coding region of the dystrophia myotonica-protein kinase (*DMPK*) gene in DM1 cause an aggregation of CUG transcripts, which interfere with the regulatory splicing activities of MBNL1 and CELF1 RNA-binding proteins, leading to a misregulation of the alternative splicing of several transcripts [60]. In FXTAS, 55–200 CGG repeats (compared with > 200 CGG repeats in FXS) in the 5’ untranslated region and increased expressions of the CGG-expanded transcripts are considered deleterious in neurons and glia, although the mechanisms have not been fully understood [49].

### DM1

**Fig. 12:**
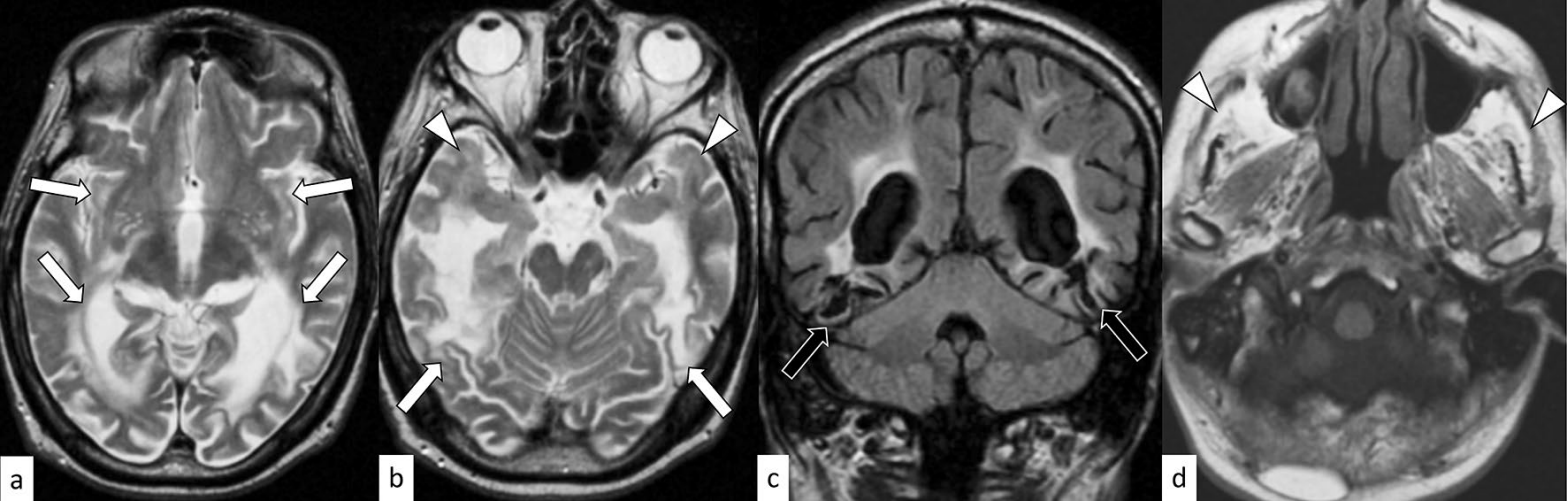
A 56-year-old man with DM1. T2-weighted axial images (**a**, **b**) show hyperintensity with atrophy in the white matter of the bilateral subcortical insular and temporal lobes (arrows). Subcortical white matter hyperintensity is also found in the bilateral temporal poles (**b**, white arrowheads). Part of the T2 hyperintensity areas shows cystic changes on FLAIR coronal image (**c**, black arrows). Severe atrophy of the bilateral temporalis and masseter muscles with fat replacement is observed on T1-weighted axial image (**d**, arrowheads)

**Fig. 13:**
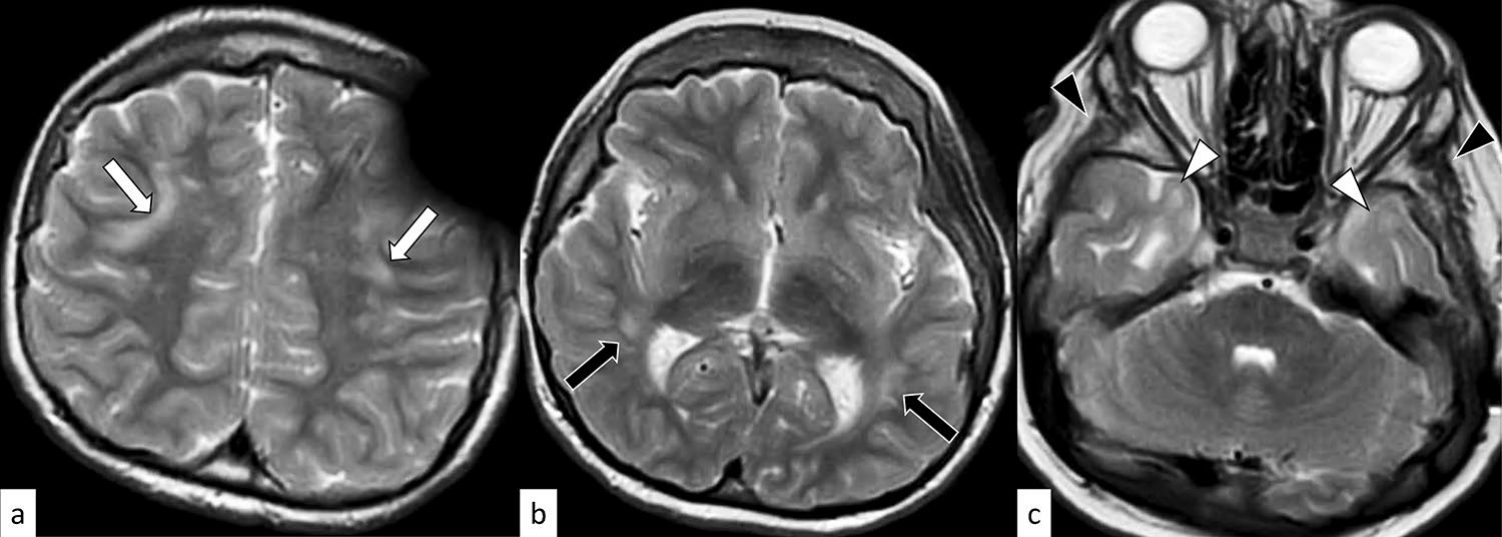
An 11-year-old boy with congenital DM1. T2-weighted axial images (**a**–**c**) show hyperintensity in the white matter of the bilateral frontal lobes (**a**, white arrows), trigone of the bilateral temporal lobes (**b**, black arrows), and the bilateral temporal poles (**c**, white arrowheads). Atrophy of the bilateral temporalis muscles is observed (**c**, black arrowheads)

DM1 is an autosomal dominant muscular dystrophy caused by an abnormal expansion of CTG repeats (≥ 50) in the *DMPK* gene on 19q13.32 [61]. DM1 can be classified based on the following five phenotypes: congenital (onset at birth to 4 weeks of age), infantile (onset between 4 weeks and 10 years of age), juvenile (onset between 10 and 20 years of age), adult/classical (onset between 20 and 40 years of age), and late-onset/mild (onset > 40 years of age) [62]. Anticipation typically occurs in the maternal transmission of DM1, and the length of CTG repeats decreases from congenital to late-onset forms [61, 62]. Congenital DM1 occurs almost exclusively via maternal transmission, and is characterized by hypotonia, respiratory failure, facial dysmorphisms (e.g., tent-shaped upper lips and a carp mouth), and mental retardation [63]. Previous studies have suggested that infantile and juvenile forms should be considered as a CNS disease rather than a muscular or systemic disease [63]. Patients with infantile and juvenile DM1 mainly develop signs of CNS dysfunction, such as mental retardation, psychopathological manifestations, fatigue, and daytime somnolence; in contrast, muscle weakness most often remains mild or may be absent during childhood or adolescence [63]. The adult form is characterized by distal muscle weakness, myotonia, facial abnormalities (“hatchet” appearance), respiratory failure, cataracts, bolding, insulin insensitivity, and cardiac arrhythmia [64]. On MRI, atrophy of the cerebral cortex; ventricular enlargement; T2-weighted hyperintensity of the frontal, insular, and temporal pole white matter; and atrophy of the masticatory muscles have been reported mostly in adults (Fig. 12) [65]. However, some reports have suggested that hyperintensity near the triangular portion of the lateral ventricles on T2WI may be characteristic of congenital and infantile forms (Fig. 13) [66].

### FXTAS

**Fig. 14:**
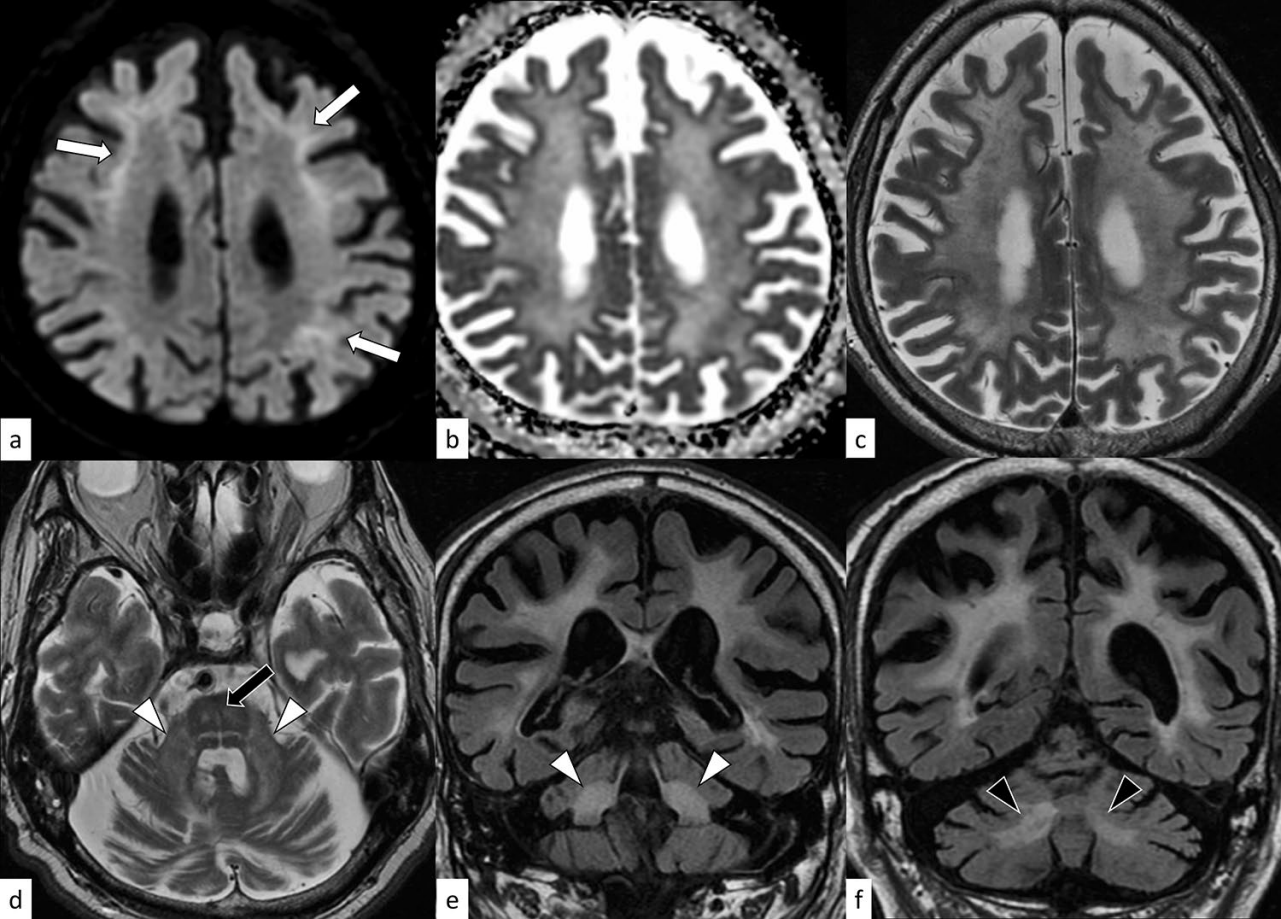
A 79-year-old man with FXTAS. DWI shows hyperintensity bands along the corticomedullary junction (**a**, arrows). ADC map (**b**) and T2-weighted axial images (**c**) show diffuse white matter hyperintensity with atrophy. The middle cerebellar peduncle sign (**d**, white arrowheads) and hot cross bun sign (**d**, black arrow) are observed on T2-weighted axial image. FLAIR coronal images show the middle cerebellar peduncle sign (**e**, white arrowheads) and paravermal hyperintensity (**f**, black arrowheads)

FXTAS is an X-linked disorder triggered by an abnormal expansion of CGG repeats (55–200) in the *FMR1* gene on Xq27.3 [50]. The mean age at onset of tremor and/or ataxia in men is 61.6 years [67]. Clinically, FXTAS is characterized by cerebellar ataxia, intention tremor, parkinsonism, cognitive decline, and peripheral neuropathy [67]. The severity of tremors and ataxia strongly correlates with the length of CGG repeats. Parkinsonism is generally mild and the effect of dopaminergic medications is limited. On MRI, the middle cerebellar peduncles (MCP) show hyperintensity on T2WI (MCP sign) in approximately 60 and 13% of affected men and women, respectively (Fig. 14) [67]. Other imaging findings of FXTAS include moderate to severe brain atrophy and non-specific white matter lesions. Although the paravermal FLAIR hyperintensity (paravermal sign) was reported as a characteristic sign for NIID and FXTAS [18], this sign can be also found in DRPLA, as mentioned above [6, 62]. Imaging overlap also occurred with hyperintensity on diffusion-weighted imaging (DWI) along the corticomedullary junctions, which was initially considered pathognomonic for NIID, although it can also be found in FXTAS, OPML, and OPDM [6, 68]. Furthermore, evidence has been accumulating that genetic analysis is essential to differentiate FXTAS from NIID, due to both clinical and imaging overlaps as well as ubiquitin- and p62-positive intranuclear inclusions on skin biopsies in either disease[69–71].

### PolyA disorders

PolyA disorders include the following diseases: cleidocranial dysplasia (CCD), holoprosencephaly type 5 (HPE5), oculopharyngeal muscular dystrophy (OPMD), *ARX* mutation-associated syndromes, blepharophimosis-ptosis-epicanthus inversus syndactyly, mental retardation with growth hormone deficiency, congenital central hypoventilation (Haddad syndrome), hand–foot–genital syndrome, and synpolydactyly [72]. Although the mechanism of polyA disorders remains unclear, it is considered to result from an unequal crossing between mispaired alleles and duplication during replication [73]. Unlike polyQ disorders, which generally exhibit neurological disorders with a wide range of phenotypes and repeat expansion lengths among patients, the polyalanine tract expansions in polyA disorders are generally smaller and more stable when transmitted between generations [73].

### CCD

**Fig. 15:**
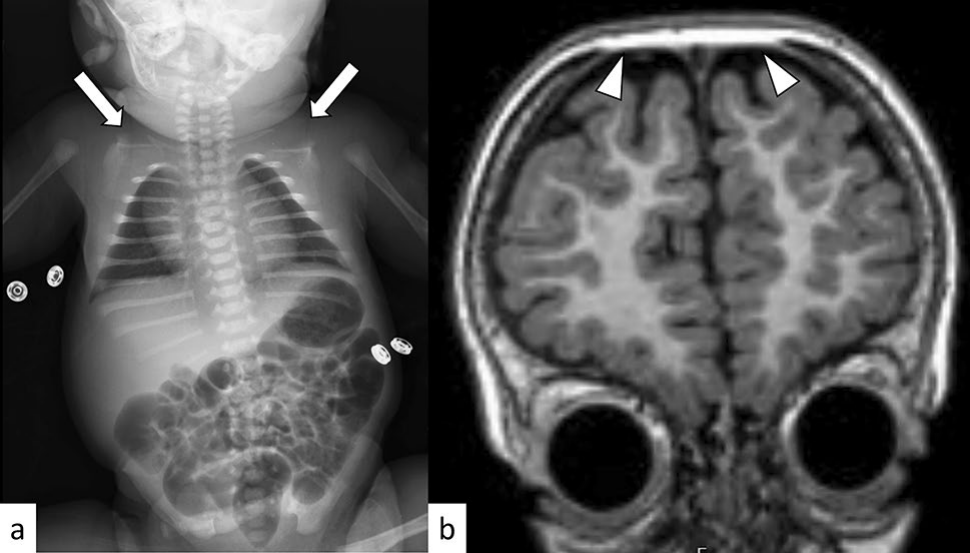
A female neonate **a** and a 2-year-3-month-old male **b** with CCD. Plain radiograph shows an absence of the bilateral clavicles (**a**, arrows). T1-weighted coronal image shows widened open anterior fontanelle (**b**, arrowheads)

CCD is an autosomal dominant disorder resulting from an abnormal expansion of GCN repeats (20–27) in the *RUNX2* gene on 6p21.1 [74]. CCD is characterized by the triad of delayed closure of the cranial sutures, hypoplastic or aplastic clavicles (Fig. 15), and dental abnormalities. Affected patients often have other skeletal problems including pes planus, genu valgum, scoliosis, and osteoporosis [74]. Intelligence is usually normal. On head and neck CT, wide open sutures, patent fontanelles, Wormian bones, arrested pneumatization of the paranasal sinuses, hypoplastic mid-facial region, relatively prognathic mandible, high-arched palate, and tooth abnormalities (impacted, crowned, and supernumerary) can be observed (Fig. 15) [74, 75].

### HPE5

HPE5 is an autosomal dominant congenital disorder caused by an abnormal expansion of GCN repeats (16–25) in the *ZIC2* gene on 13q32.3 [72]. The incidence of each type of holoprosencephaly in HPE5 is as follows: alobar (30%), semilobar (45%), lobar (15%), middle interhemispheric variants (8%), and microform (2%) [76]. Compared to other HPE types, patients with HPE5 have a more subtle common facial phenotype consisting of bitemporal narrowing, upslanting palpebral fissures, flat nasal bridge, short nose with anteverted nares, broad and deep philtrum, and a subjective appearance of large ears [77]. Other than holoprosencephaly, hydrocephalus and neural tube defects can be observed in 12% and 4% of patients with HPE5, respectively [77].

### OPMD

**Fig. 16:**
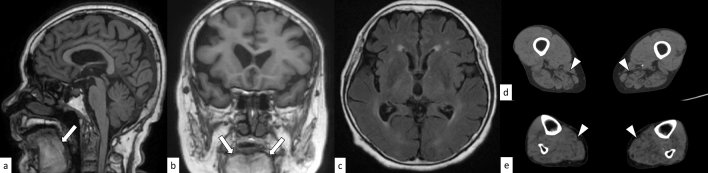
A 76-year-old woman with OPMD. T1-weighted sagittal image **a** and coronal image **b** show fat infiltration of the tongue (arrows). No significant abnormal finding is found in the brain (**c**: FLAIR axial image). Atrophy of the bilateral extensor muscles in the lower extremities is observed on non-enhanced CT (**d**, **e**, arrowheads)

OPMD is an autosomal dominant late-onset muscular dystrophy caused by an abnormal expansion of GCN repeats (12–17) in the *PABPN1* gene on 14q11.2 [78]. The mean age at onset is 48 years; nevertheless, younger age at onset is often observed in patients with longer GCN expansions. OPMD is characterized by slowly progressive ptosis, dysphagia, and proximal limb muscle weakness. On CT and MRI, fatty replacements of the muscles are identified in almost all symptomatic patients, and the tongue, soleus muscle, and adductor magnus muscle are the most commonly involved sites (Fig. 16) [79]. Brain abnormalities may be absent in OPMD [6]. Diseases manifesting as myopathy, ptosis, and dysarthria/dysphagia (including OPDM, DM1, mitochondrial neurogastrointestinal encephalopathy disease, as well as vocal cord and pharyngeal dysfunction with distal myopathy) can be clinical differential diagnoses of OPMD [78].

### *ARX* mutation-associated syndromes

A high degree of phenotypic heterogeneity has been observed in patients with *ARX* mutation-associated syndromes, and these syndromes can be divided into those with and without brain malformations [80]. *ARX* mutation-associated syndromes with brain malformations include the following X-linked polyA diseases: infantile epileptic-dyskinetic encephalopathy, infantile spasms, and Partington syndrome [80].

*ARX* mutation-associated infantile epileptic-dyskinetic encephalopathy is characterized by severe mental retardation, early-onset infantile spasms, and severe generalized dystonia that has a progressive course in the first years of life before reaching a plateau [80]. An expansion of the first of the four polyA tracts is observed in this phenotype. Brain MRI shows multiple small foci with cerebrospinal fluid intensity within the posterior inferior regions of the basal ganglia, with mildly enlarged ventricles [80]. PolyA expansion is found in the second polyA tract in *ARX* mutation-associated infantile spasms and Partington syndrome (X-linked mental retardation, seizures, and mild distal dystonia), and these diseases have not been associated with brain abnormalities on neuroimaging examinations.

### TRDs with unknown mechanisms

TRDs with unknown mechanisms include the following diseases: SCA8, SCA12, Huntington disease-like 2 (HDL2), NIID, OPML, and OPDM.

### SCA8

**Fig. 17:**
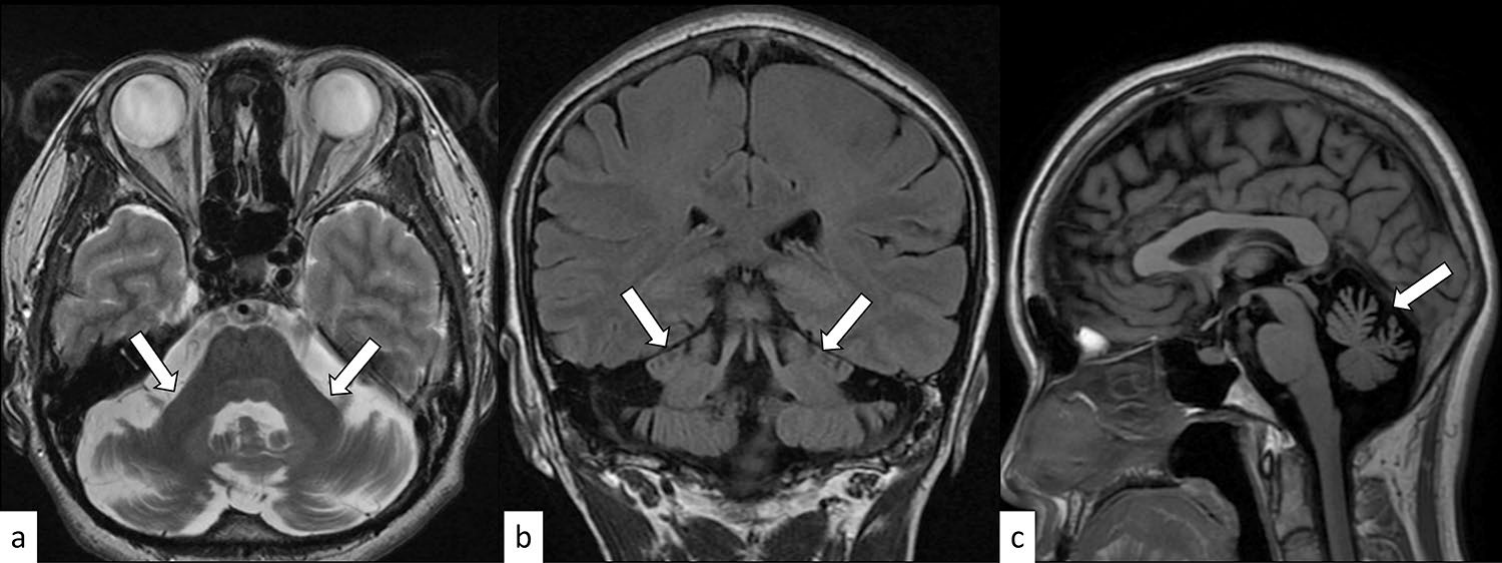
A 50-year-old man with SCA8. T2-weighted axial image **a**, FLAIR coronal image **b**, and T1-weighted sagittal image **c** show cerebellar atrophy without atrophy of the brainstem and cerebrum (arrows)

SCA8 is an autosomal dominant disorder caused by an abnormal expansion of CTG/CAG repeats in the *ATXN8* or *ATXN8OS* gene on 13q21 [81]. The typical age at disease onset is in the third to fifth decade. Patients with SCA8 exhibit slowly progressive cerebellar ataxia and a characteristic drawn-out slowness of speech. Because of low penetrance, it is sometimes challenging to diagnose SCA8 based on the family history. On MRI, global cerebellar atrophy is observed, whereas the brainstem is usually spared (Fig. 17) [82, 83]. However, the pathological significance of the increased number of triplet repeats in SCA8 has been questioned recently, given that an abnormal expansion of the triplet repeats in SCA8 has been observed in both normal controls and autopsy-confirmed multiple system atrophy [84, 85]. Roda et al. suggested that the *ATXN8* or *ATXN8OS* gene should not be evaluated in isolation as a candidate gene for spinocerebellar degenerative disease [86].

### SCA12

SCA12 is an autosomal dominant disorder triggered by an abnormal expansion of CAG repeats (51–78) in the *PPP2R2B* gene on 5q32 [87]. The mean age at onset is 34 years, and intention tremor is the most common initial presentation in SCA12. The manifestations are variable including mild cerebellar dysfunctions, tremor, gait dysfunction, pyramidal and extrapyramidal signs as well as cognitive and behavioral disturbances. MRI shows mild to moderate atrophy of the cerebellum (with vermis predominance) and cerebral cortex as well as ventriculomegaly with or without subcortical white matter changes [87]. Cerebellar atrophy (34.7%), cerebral atrophy (16.3%), or concurrent cerebral and cerebellar atrophy (34.7%) can be observed [88]. The brainstem usually shows no degenerative changes.

### HDL2

HDL2 is an autosomal dominant disorder resulting from an abnormal expansion of CTG repeats (≥ 40) in the *JPH3* gene on 16q24.2 [89, 90]. The mean age at onset is 41 years [91]. HDL2 is characterized by manifestations such as chorea, dementia, and oculomotor abnormalities; moreover, patients later exhibit a rigid and bradykinetic state with worsening dystonia [89]. Patients with HDL2 generally present with greater expression of a parkinsonism, less dysarthria, and relatively preserved oculomotor function compared to those with Huntington disease, although no significant difference in the incidence of chorea was reported between HDL2 and Huntington disease [89]. On MRI, atrophy of the caudate nuclei and putamina was frequently observed in HDL2, similar to Huntington disease [91]. Hyperintense putaminal rims on T2WI have been described in patients with HDL2 [92]. Anderson et al. reported that thalamic volume was significantly smaller in patients with HDL2 than in those with Huntington disease [93].

### NIID

NIID, an autosomal dominant, slowly progressive neurodegenerative disorder, is caused by an abnormal expansion of CGG repeats (≥ 66) in the *NOTCH2NLC* gene on 1q21.2 [6, 90]. Traditionally, NIID has the following three forms, depending on the age of onset: infantile, juvenile, and adult forms; in addition, NIID can be divided into sporadic or familial forms [94]. However, it should be noted that CGG repeats in *NOTCH2NLC* gene have been mainly reported in adult patients in Japanese and Chinese descent. Sikora et al. reported that CGG repeats in *NOTCH2NLC* gene was rare in the European patients with NIID [95]. Furthermore, Jedlickova et al. reported that CGG repeats were not expanded and skin biopsy was negative in an infantile patient with NIID [96] Clinically, key phenotypic features of infantile-onset NIID include the following: although the early development is normal, the disease onset is abrupt and the progression is rapid; initial symptoms almost always involve unsteady gait associated with cerebellar dysfunction; and in the later stage, cerebellar decline, progressive pseudobulbar or bulbar palsy, peripheral neuropathy, hypotonia, and severe psychosocial regression are observed [97]. The most common causes of death in patients with NIID include respiratory infection and respiratory failure. Sporadic adult-onset NIID is almost always characterized by dementia (94.7%) and frequent autonomic impairment resulting in miosis and bladder dysfunction. In contrast, familial adult-onset NIID is characterized by muscle weakness (100%), followed by sensory disturbance, miosis, bladder dysfunction, and dementia [94].

**Fig. 18:**
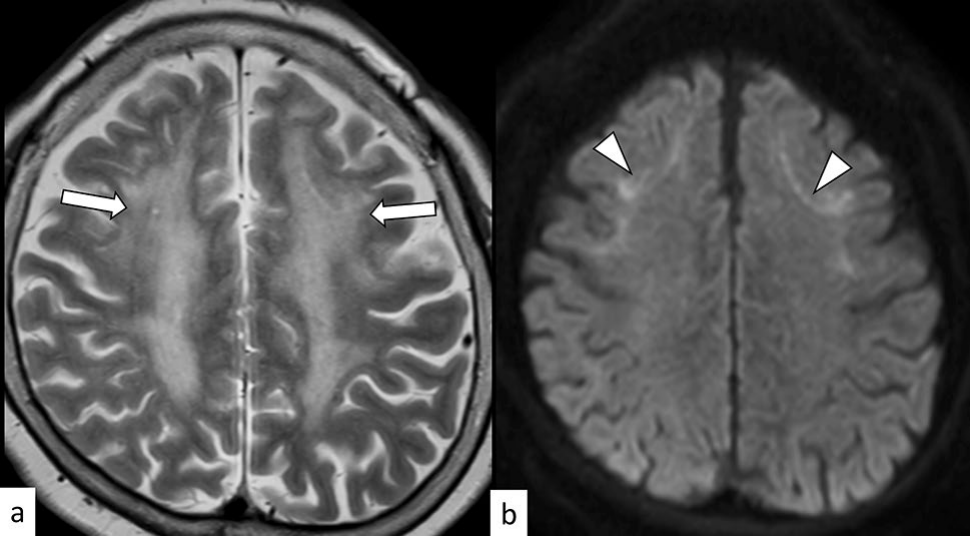
A 59-year-old woman with NIID. T2-weighted axial image shows hyperintensity in the white matter with atrophy (**a**, arrows). DWI shows hyperintensity bands along the corticomedullary junction (**b**, arrowheads). The paravermal FLAIR hyperintensity, which can be frequently observed in NIID, is not present in this case (not shown)

On MRI, abnormal hyperintensity bands on DWI along the corticomedullary junction are characteristic of adult-onset NIID, although other diseases (i.e., FXTAS, OPML, and OPDM) may also demonstrate this sign (Fig. 18) [90, 91]; in addition, the frequency of this sign in infantile- and juvenile-onset NIID is unclear. The paravermal FLAIR hyperintensity (paravermal sign) is frequently observed in NIID [19]. It is worth noting that paravermal sign can appear before other imaging findings and be the only neuroimaging clue to NIID diagnosis [98–100]. The MCP sign can also be observed in NIID. Acute encephalopathy with cortical swelling, hyperintensity on T2WI, DWI, and apparent diffusion coefficient maps with contrast enhancement have been reported in adult-onset NIID, thereby mimicking mitochondrial encephalomyopathy, lactic acidosis, and stroke-like episodes [101, 102]. In infantile-onset NIID, early-onset cerebellar atrophy is observed [97].

### OPML

**Fig. 19:**
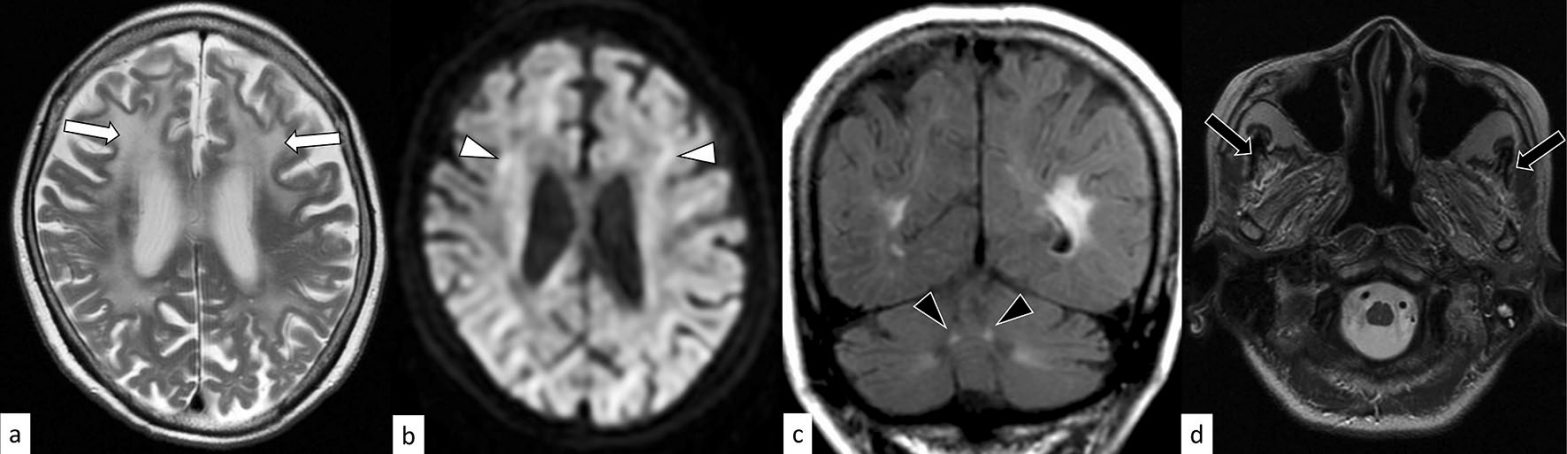
A 60-year-old woman with OPML. T2-weighted axial image shows hyperintensity in the white matter with atrophy (**a**, white arrows). DWI shows hyperintensity bands along the corticomedullary junction (**b**, white arrowheads). FLAIR coronal image also shows paravermal hyperintensity (**c**, black arrowheads). Atrophy and fat infiltration of the bilateral masticator muscles are observed on T2-weighted axial image (**d**, black arrows). The same case with different MRI slides was evaluated in a prior study [6]

OPML is an autosomal dominant disorder caused by an abnormal expansion of CCG repeats in the *LOC642361/NUTM2B-AS1* gene on 10q22.3 [6]. Given that OPML has only recently been reported, the available clinical and radiological information is limited. Frequent clinical findings of OPML include ptosis, restricted eye movements, dysphagia, dysarthria, and diffuse limb muscle weakness. On MRI, brain atrophy, T2 hyperintensity in the white matter, and DWI hyperintensity bands along the corticomedullary junction have been reported [92]. Furthermore, FLAIR hyperintensity in the paravermal area was observed in our case (Fig. 19).

### OPDM

OPDM is an autosomal dominant or autosomal recessive disorder caused by an abnormal expansion of CGG repeats in the *LRP12, GIPC1, NOTCH2NLC,* and *RILPL1* genes on 8q22.3, 19p13.12, 1q21.2, and 12q24.31, respectively. The mean age at onset is 22 years [103]. OPDM is characterized by adult-onset ptosis, ophthalmoplegia, weakness of facial and distal limb muscles, dysphagia, and dysarthria. Muscle biopsy shows myopathic changes with rimmed vacuoles. Durmus et al. reported frequent early respiratory involvement while the patients were still ambulant in OPDM, which was considered atypical for other myopathies [103]. On MRI, patients with OPDM secondary to *NOTCH2NLC* gene mutation (a common causative genetic mutation in NIID) may show DWI hyperintensity bands along the corticomedullary junction and the MCP sign [68], whereas OPDM secondary to *LRP12* gene mutation did not show any abnormal signal intensities or atrophic changes in the case presented by Ishiura et al. [93] as well as 32 case series by Kumutpongpanich et al. [104]

## Conclusion

We reviewed the clinical and neuroimaging features of TRDs. Although therapies for TRDs mainly focused on alleviating symptoms, recent advances in genetic research offer hope for the development of curative therapies. The pre-onset prediction of TRD in at-risk individuals is also becoming possible. Therefore, an early diagnosis of TRDs through clinical and imaging approaches is important and may contribute to appropriate medical interventions for patients and their families.
